# Air‐Stable Lithiation of MoS_2_ for Direct‐Bandgap Multilayers

**DOI:** 10.1002/smsc.202500186

**Published:** 2025-06-23

**Authors:** Qi Fu, Yichi Zhang, Jichuang Shen, Siyuan Hong, Jie Wang, Chen Wang, Jingyi Shen, Wei Kong, Guolin Zheng, Jun Yan, Jie Wu, Changxi Zheng

**Affiliations:** ^1^ School of Physics Research Center for Industries of the Future Department of Physics School of Science Zhejiang University Westlake University Hangzhou Zhejiang 310024 P.R. China; ^2^ School of Engineering School of Materials Science and Engineering Westlake University Zhejiang University Hangzhou Zhejiang 310024 P. R. China; ^3^ Instrumentation and Service Center for Physical Sciences Westlake University Hangzhou Zhejiang 310024 P. R. China; ^4^ Anhui Provincial Key Laboratory of Low‐Energy Quantum Materials and Devices High Magnetic Field Laboratory HFIPS Chinese Academy of Sciences Hefei Anhui 230031 P. R. China; ^5^ School of Engineering Westlake University Hangzhou Zhejiang 310024 P. R. China; ^6^ Department of Physics University of Massachusetts Amherst Amherst Massachusetts 01003 USA; ^7^ Research Center for Industries of the Future Department of Physics School of Science Westlake University Hangzhou Zhejiang 310024 P. R. China

**Keywords:** 2D materials, band structures, lithium intercalation, photoluminescence enhancement, transition metal dichalcogenide

## Abstract

Due to its sizable direct bandgap and strong light‐matter interactions, the preparation of monolayer MoS_2_ has attracted significant attention and intensive research efforts. However, multilayer MoS_2_ is largely overlooked because of its optically inactive indirect bandgap caused by interlayer coupling. It is highly desirable to modulate and decrease the interlayer coupling so that each layer in multilayer MoS_2_ can exhibit a monolayer‐like direct‐gap behavior. Herein, the nanoprobe‐controlled fabrication of Li_x_MoS_2_‐based multilayers is demonstrated, exhibiting a direct bandgap and strong photoluminescence emission from tightly bound excitons and trions. The fabrication of Li_x_MoS_2_ multilayers is facilitated by the newly developed Li‐ion platform, featuring tip‐induced Li intercalation, doping patterning with a spatial resolution of 517 nm, air stability, and rewritability. Ultralow frequency Raman characterizations reveal that controlled Li intercalation effectively transforms multilayer MoS_2_ into the stack of multiple monolayers, leading to a 26‐fold enhancement of photoluminescence compared to a monolayer. The intercalation result is different from existing observations of transforming MoS_2_ multilayers into metallic phases. This work not only provides a highly controllable Li‐ionic engineering platform for studying Li‐material interactions and developing novel ionic electronics but also offers an intriguing direct‐bandgap semiconductor for optoelectronic applications.

## Graphical abstract

This atomic‐force‐microscopy‐guided ionic modulation enables rewritable, air‐stable direct‐bandgap semiconductor fabrication of multilayer MoS_2_ without inducing metallic phase transition, unlocking monolayer‐like optoelectronic functionalities of multilayer MoS_2_. Consequently, this method is applicable to the development of various novel electronics with strong light‐matter interactions.

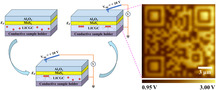

## 1 Introduction

Van der Waals (vdW) transition metal dichalcogenides (TMDCs), such as MoS_2_, WS_2_, and WSe_2_, undergo a remarkable transition from an indirect to a direct bandgap when thinned down to monolayer thickness.^[^ ^1^, ^2^, ^3^ ^]^ This direct bandgap is a pivotal property for the intensive interest in the field of 2D materials, as it enables strong light‐matter interactions and intense photoluminescence (PL).^[^ ^4^ ^]^ Owing to the enhanced Coulomb interactions and spin‐orbital coupling, the PL of monolayer TMDCs is characterized by tightly bound excitons and trions, as well as a valley degree of freedom, which are essential for exploring quantum phenomena and the development of next‐generation optoelectronics.^[^ ^5^, ^6^, ^7^, ^8^, ^9^ ^]^

To this end, the community of 2D materials has primarily focused on developing technologies for preparing monolayer TMDCs over the years. These technologies include mechanical exfoliation, chemical vapor deposition (CVD), chemical exfoliation, and laser ablation.^[^ ^10^, ^11^, ^12^, ^13^, ^14^, ^15^, ^16^ ^]^ Meanwhile, recent studies have demonstrated intense PL in bulk TMDCs after the intercalation of large organic molecules into their vdW gaps.^[^ ^17^, ^18^ ^]^ Given these findings, it is of great importance to explore whether intense PL can be achieved in Li‐intercalated MoS_2_ multilayers, considering the small size of Li ions for fast interlayer motion and the broad applications of Li‐ion‐based technology.^[^ ^19^, ^20^, ^21^ ^]^ Unfortunately, based on extensive experimental characterizations from Li‐assisted chemical exfoliation and Li‐ionic gating, it appears that intense PL and Li intercalation are incompatible.^[^ ^22^, ^23^ ^]^ This incompatibility arises because Li intercalation introduces a significant amount of n‐type doping, which quenches PL and can even transform semiconducting TMDCs into the metallic 1 T phase at substantial concentration.^[^ ^24^, ^25^, ^26^ ^]^

In this article, we report the achievement of intense PL composed of excitons and trions in multilayer MoS_2_ after Li intercalation. For clarity, we denote Li‐intercalated MoS_2_ multilayer as Li_x_MoS_2_ from here on. The Li intercalation is performed on our newly developed Li‐ionic platform, which consists of exfoliated MoS_2_ thin flakes deposited on a lithium‐ion conducting glass‐ceramics (LICGC) substrate and covered by a 27‐nm Al_2_O_3_ thin film. Unlike prevalent solution‐based doping technologies,^[^ ^10^, ^14^, ^15^, ^22^, ^26^ ^]^ here the Li intercalation is achieved in a dry environment. Particularly, the Li intercalation can be locally controlled for doping patterning by an atomic force microscopy (AFM) cantilever, using a bias voltage applied either to the backside of the LICGC substrate or to the cantilever itself, without involving any electrolytes or chemicals. Based on combined characterizations using AFM, PL, and Raman spectroscopy, the strong PL in multilayer Li_x_MoS_2_ is attributed to the isolation of interlayer coupling by Li intercalation and the reduction of Li‐induced n‐type doping by the LICGC substrate beneath after its Li depletion. In addition to strong PL, our unique Li‐ionic platform features on‐demand doping patterns with submicrometer resolution, tunable carrier density, air stability, and rewritability. Our results not only provide a unique platform for material fabrication and the development of doping patterned electronics, but also further the understanding of Li interactions with van der Waals (vdW) materials.

## 2 Results and Discussion

### 2.1 Design Concept of a Li Ionic Platform for Doping Patterning using Nanoprobe

**Figure 1:**
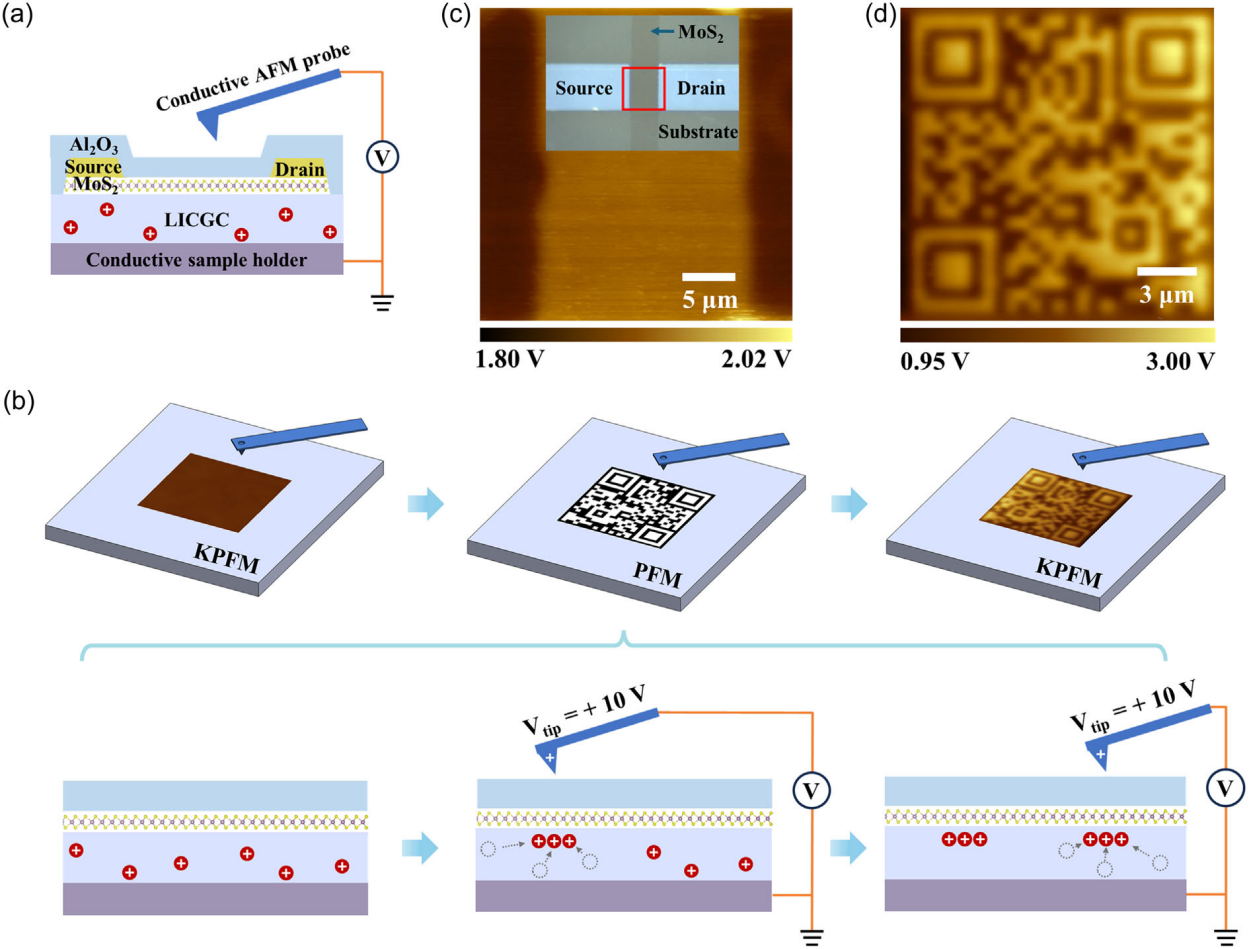
Concept of doping patterning electronics. a) Schematic of the experimental setup. b) Fabrication steps for doping patterning using KPFM and PFM. c) KPFM image of an Al_2_O_3_/MoS_2_/LICGC device with a monolayer MoS_2_ film. Inset: Optical image of the device, with the red box indicating the KPFM measurement region. d) KPFM image of a QR code doping pattern written via PFM lithography.

**Figure** **1**a presents a schematic illustration of the structure and setup for Li dopant patterning and visualization by using AFM and its accompanied techniques, including piezoresponse force microscopy (PFM) and Kelvin probe force microscopy (KPFM).^[^ ^27^, ^28^, ^29^ ^]^ As shown, the technique has a simple structure where a 2D material (MoS_2_) is deposited on an LICGC substrate and covered by a layer of oxide dielectrics.^[^ ^30^ ^]^ Such kind of structure is denoted as Al_2_O_3_/MoS_2_/LICGC from here on. Throughout the scanning procedure, either the reverse side of the LICGC or the AFM cantilever is electrically grounded. Both work equally well.

The detailed process for doping patterning in an arbitrary region is given in Figure 1b. Firstly, the channel region of interest on the prefabricated 2D device is determined by AFM scanning, and the original electronic property of this region is observed using KPFM. Second, doping patterns are rendered into the selected region by translating the designed voltage pattern of AFM tip to the sample surface using PFM lithography technique that has been widely used in ferroelectric materials.^[^ ^31^ ^]^ During the PFM lithography process, applying a positive voltage to the AFM tip causes Li ions to migrate toward the interface between the monolayer MoS_2_ and the substrate. This results in the formation of a localized electric double layer (EDL) in the substrate. Afterwards, the doping patterns can be confirmed by KPFM visualization.

**Figure 2:**
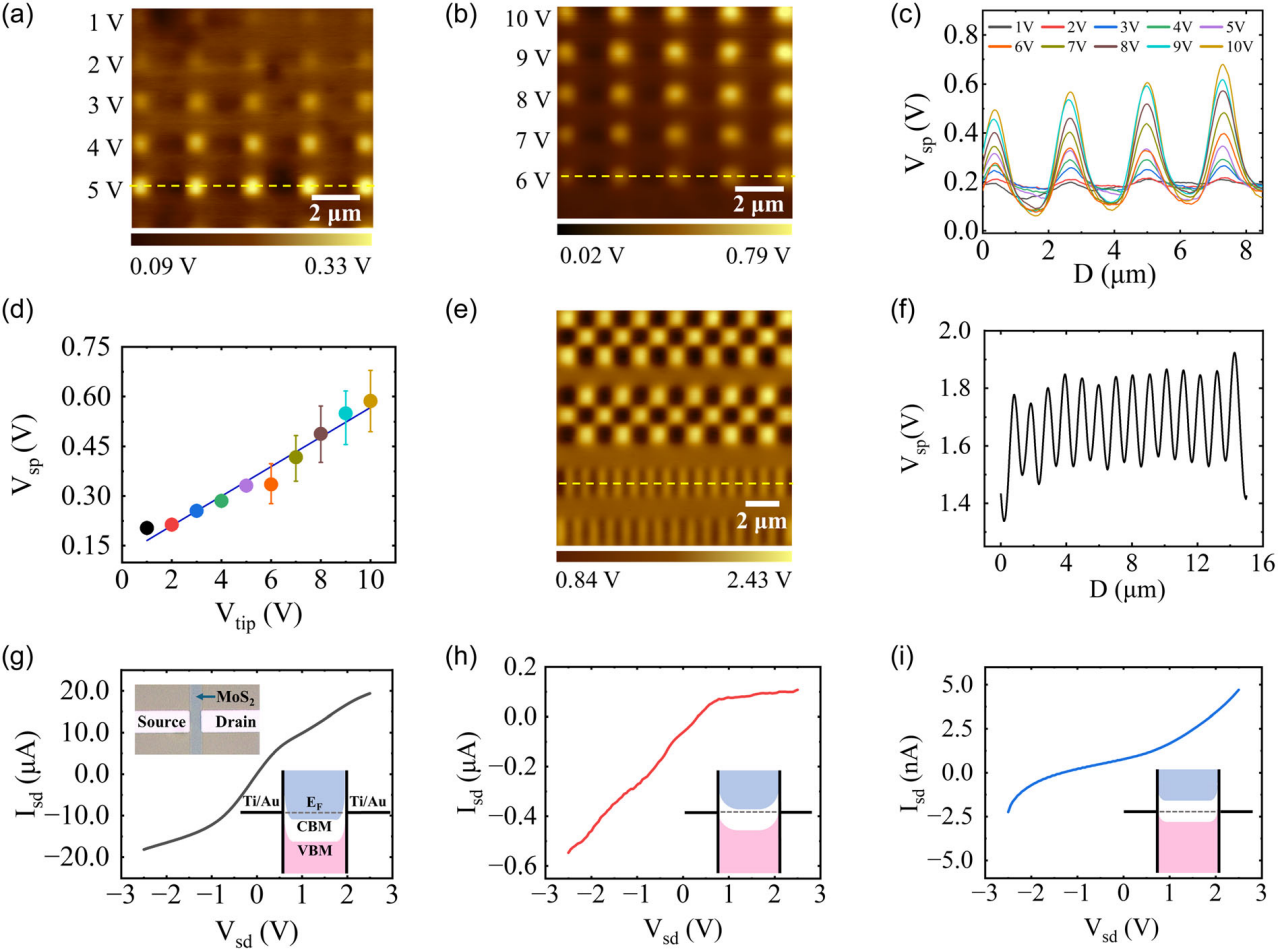
Modulations of doping patterns and device characteristics. a–b) KPFM images of tip voltage series ranging from +1 V to +10 V in 1 V steps. c) Line profiles of surface potential ($V_{\text{sp}}$) patterns from a,b). Yellow dashed lines in a),b) mark typical extraction paths. d) Surface potential peak values (from c) versus PFM lithography voltage; blue line indicates linear fit. e) KPFM image of doping patterns written via PFM lithography at varying tip voltages. f) Surface potential line profile along the yellow dashed line in e). Averaged full width at half maximum (FWHM) 517 nm defines doping resolution. g–i) Current–voltage (I‐V) evolution of an Al_2_O_3_/MoS_2_/LICGC device: g) pristine state, h) after −5 V PFM lithography, and i) after −8 V PFM lithography. Inset: Optical microscopy image of the device.

Figure 1c presents a KPFM image of a MoS_2_ device with Ti/Au contacts covered by a 27 nm thick alumina (Al_2_O_3_) film. The optical microscopy image of the device is displayed in the Inset. Raman and photoluminescence (PL) spectra confirm the monolayer thickness of the MoS_2_ film,^[^ ^1^, ^2^, ^32^ ^]^ which was grown by CVD, see Methods and Figure 1, Supporting Information. Following the growth, the MoS_2_ film is carefully transferred onto the LICGC substrate using a wet chemical process. Afterward, the device is fabricated and encapsulated with an Al_2_O_3_ film via atomic layer deposition (ALD), as described in the Methods section. Given the exceptional dielectric properties of Al_2_O_3_, the overlaying thin film does not hinder the long‐range Coulombic interactions between the AFM tip and the device, thus enabling the capture of a high‐resolution KPFM image of the device.^[^ ^33^ ^]^ The surface potential of the Ti/Au contact, as shown in Figure 1c, is ≈200 mV lower than that of monolayer MoS_2_. This implies that the Fermi level of the Ti/Au contact is positioned below that of MoS_2_. Consequently, the work function of MoS_2_ is smaller than that of Ti/Au, aligning with the findings reported in the literature.^[^ ^34^ ^]^ It should be noted that the surface of LICGC is not well grounded during KPFM measurements due to its electrical insulation, resulting in a floating potential that hinders the precise measurement of the work function of the device on top. However, the relative surface potential differences between different components on the surface, such as the Ti/Au contacts and the MoS_2_ channel here, can be measured accurately. Prior to measuring the samples, the work function of each AFM cantilever is calibrated using an Au‐Si‐Al sample, as shown in Figure 2, Supporting Information.

**Figure 3:**
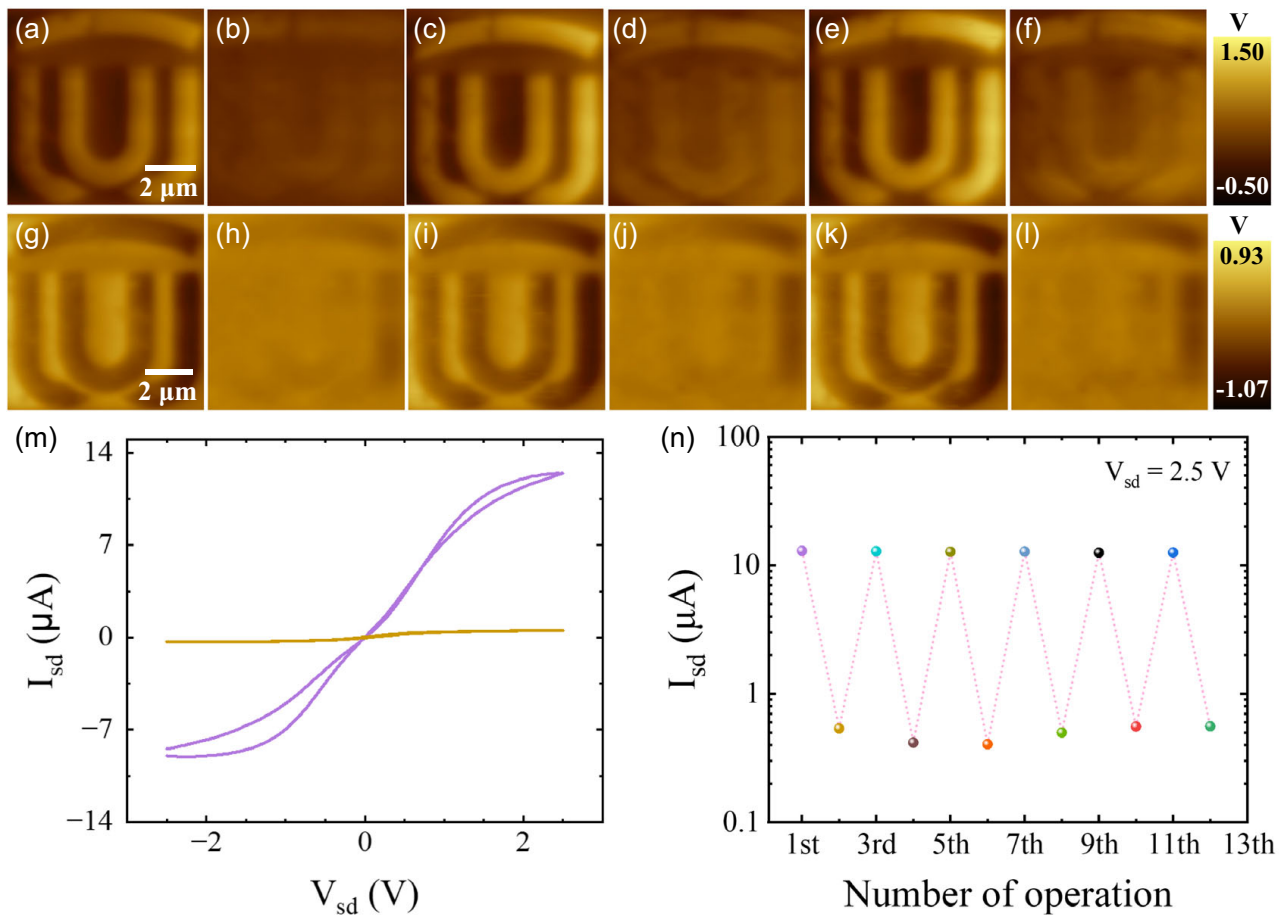
Erasable and repeatable ionic doping. a–f) A doping pattern written with +8 V and erased with −3.4 V over three cycles. Scale bars in a) applies to b–f). g–l) The same doping pattern written with −8 V and erased with +2.5 V over three cycles. Scale bar in g) applies to h–l). m) *I*–*V* curve measurements of an Al_2_O_3_/MoS_2_/LICGC device, showing two resistance states switched by PFM lithography applied to the channel. n) Repeated source–drain current values ($I_{\text{sd}}$) at $V_{\text{sd}}\;=+2.5\text{ V}$ using PFM switching.

**Figure 4:**
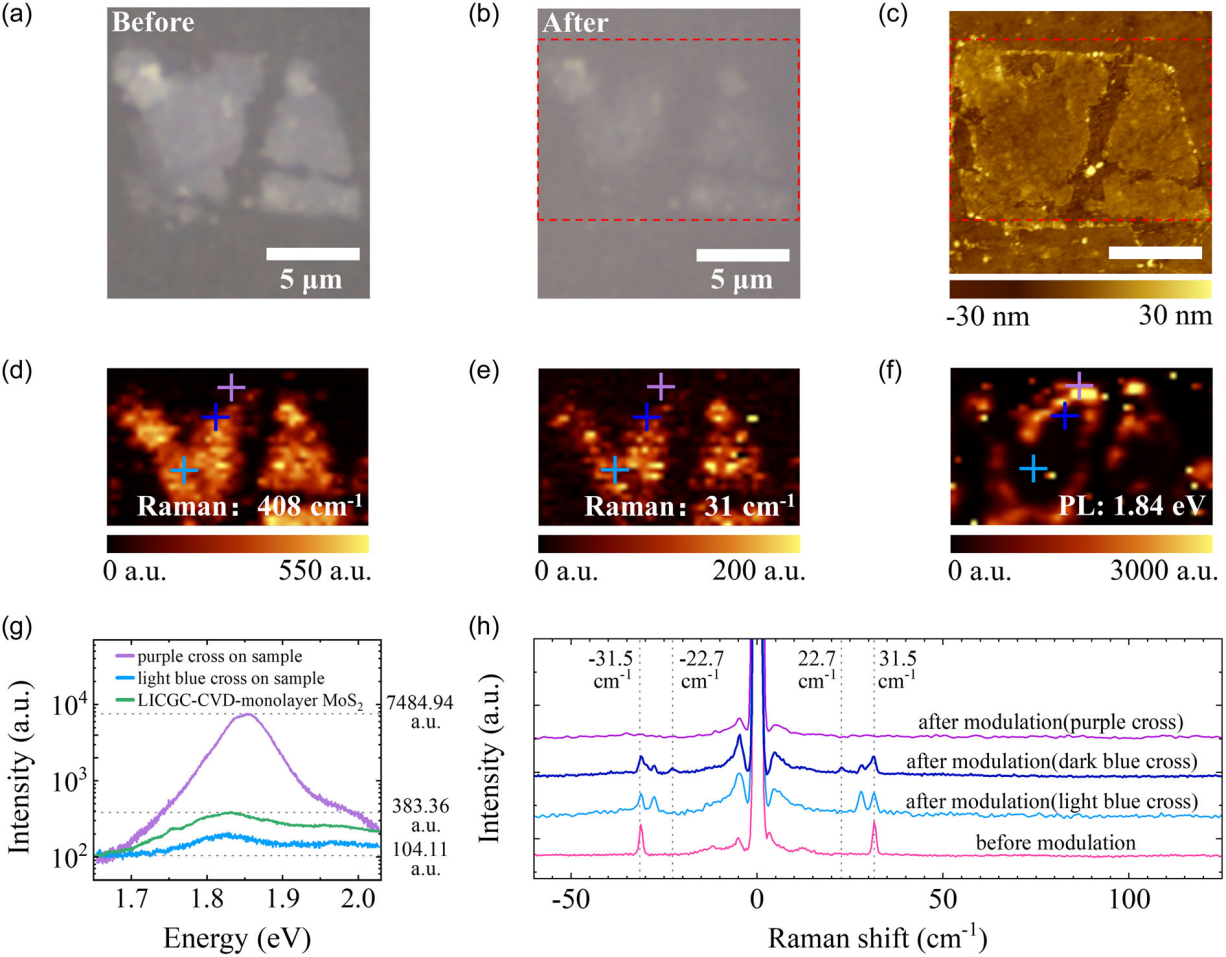
Impact of AFM tip‐induced Li intercalation on optical properties of Li_x_MoS_2_. a,b) Optical microscopy images of mechanically‐exfoliated MoS_2_ flakes a) before and b) after Li intercalation via AFM tip modulation. c) AFM topography of the intercalated flakes (scale bar: 5 μm). d–f) Correlated Raman and photoluminescence (PL) mappings of the region highlighted in b) and c). Crosses mark the locations for single‐point spectral acquisition. g) PL spectra from the crosses in f) and a) CVD‐grown monolayer MoS_2_ transferred onto a LICGC substrate. h) ULF Raman spectra at the crosses in d),e), and for pristine multilayer MoS_2_ flake (left flake in a).

Since the work function difference at the interface buried by the Al_2_O_3_ film can be clearly imaged using KPFM, we further explore the capability of moving Li ions in LICGC substrate by applying voltage to the conductive AFM tip. For this purpose, PFM lithography is employed, as it has been widely used to write arbitrarily designed voltage patterns onto ferroelectric surfaces.^[^ ^31^ ^]^ For comparative analysis, PFM lithography is performed on both the bare LICGC substrate and the one covered by a 27 nm Al_2_O_3_ film. Note that LICGC covered by an Al_2_O_3_ film without MoS_2_ is denoted as Al_2_O_3_/LICGC from here on. A voltage pattern of a Quick Response (QR) code, as shown in Figure 3a, Supporting Information, is written onto both types of samples. Figure 1d displays the KPFM image of the surface of LICGC covered by the Al_2_O_3_ film after writing the QR code, and the corresponding AFM topography is shown in Figure 3b, Supporting Information. The contrast observed within the KPFM image corresponds to the distinct doping regions, which are precisely defined by the PFM tip voltage. In contrast, no distinct KPFM contrast is observed on the surface of the bare LICGC, as seen in Figure 4, Supporting Information. The absence of contrast is likely due to the reactivity of Li ions with ambient air components, whereas the Al_2_O_3_ film can effectively protect them from such interactions.

### 2.2 Parameters for Doping Patterning and Electronic Behaviors

**Figure 5:**
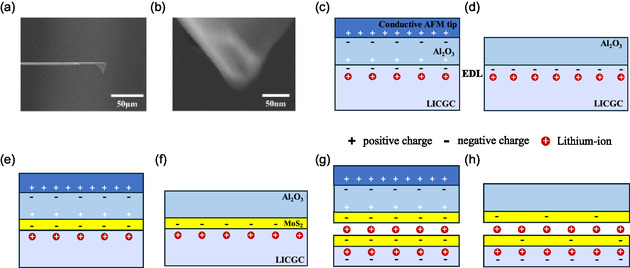
Mechanism of doping patterning and Li intercalation. a) SEM image of an AFM cantilever. b) Zoomed‐in SEM image of the AFM tip. c) Schematic of charge distribution during contact with an AFM tip at positive voltage during PFM lithography. d) Residual EDL after tip withdrawal. e) Schematic of charge distribution in an Al_2_O_3_/MoS_2_/LICGC device with monolayer MoS_2_ upon contact with a positively biased tip. f) Residual EDL after tip withdrawal. g) Schematic of an Al_2_O_3_/MoS_2_/LICGC device with Li intercalation and bilayer/multilayer MoS_2_ upon contact with a positively biased tip. The negative charges at the bottom are caused by Li depletion. h) Residual intercalated Li ions after tip withdrawal, illustrating the combined doping effects of intercalated Li and the LICGC substrate with Li depletion layer on MoS_2_ layers.

To further explore the PFM doping mechanism in Al_2_O_3_/MoS_2_/LICGC, doping arrays were generated by varying the tip voltage in a series from +1 V to +10 V, with steps of 1 V, see **Figure** **2**a,b. The corresponding line profiles of the dopant rows defined by the tip voltage series are presented in Figure 2c. As shown, the electron doping level increases as the tip voltage rises. By measuring the peak values of potential differences as a function of tip voltage, a linear relationship between the surface potential shift and the tip voltage is obtained (see Figure 2d). It should be noted that as the tip voltage increases, the peak value of surface potential rises, while the valley value decreases, as observed in Figure 5, Supporting Information. These results suggest that the motion of Li ions, induced by the tip voltage, is not only in the direction of LICGC substrate thickness but also along the surface, due to the sharpness of the AFM tip. Thus, the accumulation of Li ions is usually accompanied by Li depletion in the vicinity, generating reverse doping. Figure 2e presents the doping patterns, including a chessboard and fine bars, created by alternating the tip voltage from positive to negative, see Figure 6, Supporting Information, for a PFM voltage pattern. By taking the line profile of the fine bars (Figure 2f), the spatial resolution of the doping pattern, which is around 517 nm, can be determined. All results indicate that the doping level, as well as the features and size of the doping pattern, are highly controllable in our solid‐state ionic chip.

Further confirmation of tip‐writing controlled doping can be obtained through current‐voltage (*I‐V*) curve measurements of Al_2_O_3_/MoS_2_/LICGC devices. As shown in Figure 2g, the MoS_2_ is electron‐doped after being deposited on the LICGC substrate due to the presence of Li ions on the substrate surface and the native doping induced by CVD growth process. However, the *I‐V* curve exhibits Schottky diode‐like characteristics after PFM writing with a negative tip voltage (Figure 2h). The Schottky diode‐like *I‐V* curve is caused by the work function difference between the lightly doped MoS_2_ monolayer and the Ti/Au electrodes, as well as the asymmetric contact areas of the source and drain electrodes, which is due to the precision limits of general microfabrication. After further writing using higher negative voltage, the Al_2_O_3_/MoS_2_/LICGC device eventually exhibits insulator‐like properties (Figure 2i). The results of electronic devices suggest that negative tip voltage would expel Li ions away from MoS_2_ film and remove electron dopant. Moreover, as shown in Figure 7, Supporting Information, the tip‐induced doping is air stable due to the protection of Al_2_O_3_ thin film.^[^ ^35^ ^]^

### 2.3 Rewritable Doping Patterns on Demand

Thus far, our results demonstrate that tip voltage patterning is an effective method for fabricating electronic devices with precise control over doping levels and regions. **Figure** **3**a,g presents KPFM images depicting a logo created by doping patterning, utilizing +8 V and –8 V tip voltages, respectively. The corresponding voltage maps are shown in Figure 8, Supporting Information. As observed, the uniform contrast in the images indicates that a consistent level of doping has been achieved. After applying –3.4 V to the top row and +2.5 V to the bottom row regions, the doping patterns are nearly erased, leaving only a faint signal, as shown in Figure 3b,h. This process is repeatable, and here we illustrate its repeatability over three cycles, as visualized by KPFM in Figure 3c–f,i–l. It should be noted, though, that there are residual signals which intensify with each successive cycle.

The exceptional repeatability of our solid‐state ionic chip paves the way for the fabrication of memory electronics. As depicted in Figure 3m, both high and low resistance states can be induced through tip voltage writing, resulting in an order of magnitude change in channel current. The device's performance is reproducible, and Figure 3n demonstrates multiple consecutive repetitions here. The extra rewritable figures of the monolayer MoS_2_ channel resistance could be seen in Figure 13, Supporting Information.

### 2.4 Nanoprobe Fabrication of Multilayer Li_x_MoS_2_ with a Direct Bandgap

Li‐ion doping, in contrast to traditional ion implantation for dopant patterning, modifies not only the Fermi level of the material but also has the potential to decouple the interlayer interactions of vdW materials through Li intercalation.^[^ ^24^ ^]^ A prime example is the chemical exfoliation of vdW materials facilitated by Li intercalation.^[^ ^14^, ^15^ ^]^ This ionic doping characteristic differs from the electronic modulation effects observed in 2D electronics due to electrostatic gating.^[^ ^36^ ^]^ Specifically, the conventional metal‐insulator‐semiconductor‐structure‐based electrostatic modulation effects are constrained by the breakdown electric field of dielectric layers, imposing significant limitations on achievable carrier doping concentrations in semiconductors. Compared with traditional methods, ion‐doping modulation systems not only allow for a wide range of carrier density modulation in materials but also induce structural changes through ion migration.

**Figure** **4**a displays the optical microscope image of two mechanically‐exfoliated MoS_2_ thin flakes, which have been deposited on a LICGC substrate and coated with a 27 nm Al_2_O_3_ film via ALD (refer to the Methods section). Following PFM writing at +10 V to the two flakes, their optical contrast becomes weaker and even vanishes at edge regions, as shown in Figure 4b. The distribution of ionic current during this process has been shown in Figure S14, Supporting Information. The change in optical contrast is a strong indicator of Li intercalation, which normally starts from edges and progresses toward the center, as observed by other groups.^[^ ^10^, ^19^, ^26^ ^]^ Figure 15, Supporting Information, could illustrate this process schematically. In order to further understand this optical phenomenon, we established a simple model of optical contrast before and after Li intercalation into MoS_2_, for details, please refer to Supporting Information Section S3. The AFM topography of the flakes after Li intercalation is presented in Figure 4c. The surface height variations between the edges (transparent) and the central regions (nontransparent) are attributed to Li intercalation, as shown in Figure 9, Supporting Information. Furthermore, the thickness variation of the other MoS_2_ flakes of different width in the PFM lithography scanning direction before and after Li intercalation has been shown in Figure 10, Supporting Information. However, precise measurement of the height difference via AFM is challenging due to the roughness of the LICGC surface. The increase in interlayer distance induced by Li intercalation can be confirmed by the cross‐sectional transmission electron microscopy (TEM) results presented in Figure 17, Supporting Information.

Raman and PL mappings were carried out to investigate the two flakes (see Figure 4 and Methods for more details). Figure 4d,e show the intensity mappings of the intra‐ and interlayer Raman modes, located at 408 and 31 cm^−1^, respectively.^[^ ^37^ ^]^ It should be noted that these optical characterizations were performed in air, demonstrating the air stability of our Li‐ionic platform. As shown, the Raman maps exhibit strong intensities in the central opaque areas in Figure 4b. In contrast, the PL maps of the two flakes (shown in Figure 4f) indicate intense optical emission on the edges of the flakes. The results clearly suggest that the AFM tip‐induced Li intercalation effectively enhances the PL of multilayer MoS_2_. The difference in Raman intensities shown in Figure 4d, e can be attributed to the factors including optical interference and the change in electronic structure of MoS_2_.

Figure 4g presents the PL spectra from representative locations marked by purple and light blue crosses in Figure 4f. For clarity, the PL and Raman spectra from these regions are labeled as follows: purple‐PL, purple‐Raman, light blue‐PL, light blue‐Raman, and dark blue‐Raman (Figure 4d–h). Both PL spectra exhibit strong A peaks at 1.86 eV (purple) and 1.82 eV (light blue), respectively.^[^ ^4^, ^38^ ^]^

For comparison, Figure 4g also includes the PL spectrum of a CVD‐grown monolayer MoS_2_ film transferred onto LICGC (labeled CVD‐PL). In order to present the images in a clear and concise manner, we replaced the deep blue curve, which is similar to the light blue curve, with the green PL curve of the LICGC‐CVD‐monolayer MoS_2_ sample to provide contrast with the other curves. The CVD‐PL peak intensity is stronger than that of the light blue‐PL taken from inner flake region with lower Li intercalation. Strikingly, the purple‐PL, taken from edge region with higher Li intercalation density, shows a peak intensity exceeding CVD‐PL by 26‐fold. This dramatic PL enhancement is prominent evidence suggesting a transition from indirect to direct gap. This direct bandgap induced by intercalation and interlayer distance expansion in multilayer MoS_2_ could be demonstrated through density functional theory (DFT) calculations. For more details, please refer to the Supporting Information Section S1.

We propose that tip‐induced Li intercalation decouples interlayer interactions in multilayer MoS_2_, converting it into a vertically aligned stack of monolayers. The absence of the new Raman modes associated with the 2H‐to‐1T phase transition at ≈150, 200, or 300 cm^−1^ in the Raman spectra shown in Figure 16, Supporting Information, indicate that the MoS_2_ remains in 2H phase after Li intercalation.^[^ ^39^, ^40^, ^41^ ^]^ This contrasts with prior reports, where complete interlayer decoupling in MoS_2_ requires Li concentrations approaching 1 Li per unit cell—a regime that induces a metallic 1T phase and quenches PL entirely.^[^ ^24^ ^]^ This difference likely arises from the distinct Li intercalation dynamics between solution‐based methods and our approach.

To examine this claim, ultralow frequency (ULF) Raman spectroscopy was performed to characterize the locations indicated by the crosses in Figure 4d,e. The ULF Raman characterizations are taken in a cross‐circular polarization setup,^[^ ^42^ ^]^ focusing on the interlayer shear mode vibration that is highly sensitive to layer numbers.^[^ ^24^, ^37^, ^42^ ^]^ The pink Raman spectrum shown in Figure 4h was obtained from the MoS_2_ multilayer before AFM tip modulation. As shown, the shear mode of the pink Raman spectrum peaks at 31.5 cm^−1^, close to the value of bulk MoS_2_. Upon Li intercalation, the inner regions of the left flake (light blue‐Raman and dark blue‐Raman) exhibit Raman peaks at wavenumbers below 31.5 cm^−1^, suggesting the emergence of thinner layers due to Li intercalation even away from the edges. Notably, the dark blue ‐ Raman spectrum, taken from the region closer to the intense PL area, shows a peak at 22.7 cm^−1^, corresponding to bilayer MoS_2_.^[^ ^37^ ^]^ This Raman spectrum indicates the presence of a stack of various thin layers in this region. When moving to the region of intense PL, no shear mode is observed. Considering the appearance of intense PL, we can conclude that the intercalated Li ions effectively decouple the interlayer interactions of multilayer MoS_2_ and transform it into a stack of multiple MoS_2_ monolayers.

It is also of interest to note that the room temperature purple‐PL is peaked at 1.86 eV, which is similar to that of mechanically‐exfoliated monolayer MoS_2_, indicating limited charge doping in the sample. In the Figure 11, Supporting Information, we show low‐temperature PL where it is more straightforward to deconvolute the exciton and trion contributions. By comparing the exciton and trion energy and spectral weight, the Fermi energy of our Li_*x*_MoS_2_ is only about 15 meV.^[^ ^43^ ^]^ Based on this result, a preliminary estimation of the charge doping concentration in the system has been made: $n_{2D}\approx 3.13\;\times \;10^{12}{\text{ cm}}^{-2}$. For more details, please refer to the Supporting Information Section S2. These observations clearly indicate that the dynamics of AFM tip‐induced Li intercalation are significantly different from those of solution‐based Li intercalation.^[^ ^10^, ^21^, ^24^, ^26^ ^]^

### 2.5 Models for Doping Patterning via PFM Lithography

Based on the above results, we can try to conclude the mechanism of patterning doping and Li intercalation using PFM lithography. First, the radius of the conductive AFM tip apex was observed using scanning electron microscopy (SEM), see **Figure** **5**a. Figure 5b indicates the conductive AFM tip exhibiting nanoscale sharpness, with a radius of 25 nm.^[^ ^44^ ^]^ According to basic electromagnetic principles, a high density of positive charges accumulates on the surface of the tip apex, generating a large and localized electric field when a positive voltage is applied to the AFM tip. This effect is widely utilized in field ion microscopy and atom probe tomography.^[^ ^45^, ^46^ ^]^ When the tip voltage is fixed at *U*, the tip apex electric field *E* is inversely proportional to the tip radius *R*, following the equation $E=\;\frac{U}{R}$.

We have established a simplified model: When the AFM tip contacts the upper surface of the dielectric material and a voltage *U* is applied, a large electric field *E* is generated due to the high density of charges accumulating on the tip's surface, a result of the tip enhancement effect. According to Gauss's law, the relationship between the electric field *E* and the surface charge density *σ* at the tip apex is given by the equation $E=\;\frac{\sigma }{4\pi \varepsilon }$, where *ε* is the vacuum dielectric constant.

When the tip contacts the top surface of Al_2_O_3_, the dielectric film is polarized by the tip's electric field, inducing opposite charges on each side, see Figure 5c. Here, we consider the scenario where the tip has a positive voltage and the contact region between the tip and Al_2_O_3_ is a nanoscale flat plane. Due to the formation of charges on the Al_2_O_3_ surface, an EDL forms at the vicinal surface of the LICGC substrate because the Li ions move further into the bulk. When the AFM tip withdraws, the EDL in the LICGC remains, as the Li ions inside are static without external stimulation such as an electric field, as seen in Figure 5d.

However, in the case of Al_2_O_3_/MoS_2_/LICGC device with monolayer MoS_2_, the EDL consists of a layer of negative charges in the MoS_2_ and a complementary layer of positively charged Li ions in the LICGC, as shown in Figure 5e. Upon withdrawing the AFM tip, the additional electron dopants remain in the monolayer MoS_2_ (see Figure 5f). Conversely, when a negative voltage is applied to the AFM tip, hole dopants are introduced into the monolayer MoS_2_ to neutralize its native electron dopants. Concurrently, Li ions in the LICGC move away from the MoS_2_, forming a negatively charged layer; see Figure 12, Supporting Information. Consequently, the natively electron‐doped MoS_2_ exhibits increased resistance after the AFM tip is withdrawn, as illustrated in Supplementary Figure 12b. The model explains the results presented in Figure 2g–i.

Lastly, it should be noted that under a large positive tip voltage, Li intercalation occurs in the Al_2_O_3_/MoS_2_/LICGC system with multilayer MoS_2_, see Figure 5g,h. This occurs because a large amount of negative charge can be generated in the multilayer MoS_2_ thin flake when a large positive voltage is applied to the tip. Consequently, Li ions in the LICGC move toward MoS_2_, leading to Li intercalation starting from the edges of the flake. This model can explain the results shown in Figure 4. After withdrawing the AFM tip, the intercalated Li ions remain stationary. Alternatively, the LICGC substrate surface, with Li depletion, can provide a gating effect that partially neutralizes the electron doping induced by the intercalated Li ions, resulting in intense PL.

## 3 Conclusion

In summary, we demonstrate a controllable ionic doping method by utilizing a solid Li‐ion conductor LICGC in conjunction with a thin layer of an oxide dielectric film. The thin oxide film not only shields Li ions from the ambient environment, which is highly reactive to Li, but also permits the enhanced electric field of the AFM tip to control the motion of Li ions. The doping level and even Li intercalation can be regulated by the tip voltage, and the active region of Li‐ion accumulation can be confined by the AFM nanotip head. Particularly, the tip‐induced Li intercalation can completely screen the interlayer coupling of multilayer MoS_2_, enabling the successful transformation of multilayer MoS_2_ into vertically stacked multiple monolayers with a direct bandgap for strong light‐matter interactions, without transitioning to the metallic 1T phase.

Our study confirms the localized high charge density enhanced by the nanotip can direct the forward and backward motion of the Li ions, depending on the voltage polarity, leading to the creation or erasure of doping patterns. The doping and ion intercalation technique presented herein is distinct from the existing methods,^[^ ^25^, ^47^, ^48^ ^]^ featuring high carrier density, customizable doping patterns, rewritability, air stability, and the nanoprobe fabrication of Li‐intercalated materials with emerging phenomena. Consequently, our method is applicable to the development of various 2D electronics with strong light‐matter interactions.

## 4 Experimental Section

4.1

4.1.1

##### Solid Li‐Ion Electrolyte

The specific component information of LICGC (product AG‐01, from Ohara Corp.) was Li_2_O‐Al_2_O_3_‐SiO_2_‐P_2_O_5_‐TiO_2_‐GeO_2_, which was a solid electrolyte material exhibiting high lithium‐ion conductivity ($1\;\times \;10^{-4}{\text{ S cm}}^{-1}@25\;\text{°C}$). It belongs to the category of oxide solid and exhibits stability against air and water.^[^ ^49^ ^]^

##### 2D Materials Preparation and Transfer

Monolayer MoS_2_ films were grown using the same CVD method reported.^[^ ^50^ ^]^ Transfer of single‐layer MoS_2_ was performed using a PMMA‐based method. Initially, the PMMA solution (950 K A4, Micro Chem Corp) was spin‐coated onto the single‐layer MoS_2_/sapphire substrate. The spin‐coating was executed at a rate of 500 rpm for 5 s, followed by 2000 rpm for 55 s, and then dried for 3 min at a temperature of 80 °C. Subsequently, a potassium hydroxide (10 m, Sinopharm) solution was utilized to etch the sapphire substrate, allowing the PMMA/MoS_2_ film to float on the solution's surface. The PMMA/MoS_2_ film was then thoroughly washed with deionized water to eliminate any etchant residue. Following this, the PMMA/MoS_2_ film was carefully scooped out using a LICGC substrate and dried overnight to remove any trapped water between the MoS_2_ and the substrates. Lastly, the PMMA was removed by immersing the sample in an acetone bath and subsequently dried using compressed nitrogen gas.

##### Device Fabrication

Ultraviolet lithography (Suss MicroTec Lithography, MA/BA6 Gen4) was used to fabricate electrode patterns. Then, Ti/Au (2 nm/8 nm) was deposited by E‐beam evaporation and lift‐off with acetone. After the secondary ultraviolet lithography, O_2_ plasmas were used to etch redundant single‐layer MoS_2_. Finally, the ALD system (SENTECH Instruments GmbH, SI ALD LL) was used to deposit Al_2_O_3_ with a thickness of about 27 nm at 150 °C.

##### AFM and Electrical Characterizations

The surface potential was modulated and analyzed via PFM and KPFM measurements with Cypher ES (Oxford) at room temperature. A Ti/Ir‐coated conductive tip (Oxford, ASYELEC‐01‐R2) with a free resonance frequency of 75 kHz and a spring constant of 2.8 N m^−1^ was used. In all PFM and KPFM measurements, the used scan rate was 1.0 Hz. Based on our independently established in situ PFM modulation and electrical measurement platform, we used varnished wire to connect the electrodes fabricated on the sample device using micro‐nano processing techniques to the Keysight B2901A source measure unit through an electrical interfacing channel, enabling in situ measurements of the device electrical properties after modulated within the AFM chamber.

##### Raman and PL Characterization

Raman and PL spectra were measured at room temperature using the Witec Alpha 300 RAS Raman spectrometer equipped with 300/1800 lines per mm gratings and a 532 nm wavelength laser. The power applied to the sample was ≈3 mW for Raman and PL measurements. Scattered light was collected through a 100 × objective (Zeiss LD EC Epiplan‐Neofluar Dic 100 × /0.75). Raman and PL spectra measurement at low temperature using the Horiba and attocube equipped with 600/1800 lines per mm gratings and a 532 nm wavelength laser. The power applied to the sample was ≈3 mW for Raman and PL measurements. Scattered light was collected through a 100 × objective (LT‐APO/532‐RAMAN/0.82).

## Conflict of Interest

The authors declare no conflict of interest.

## Author Contributions

**Qi Fu**: conceptualization (equal); data curation (lead); formal analysis (equal); methodology (lead); project administration (lead); visualization (lead); writing—review and editing (lead). **Yichi Zhang**: data curation (lead); methodology (lead). **Jichuang Shen**: methodology (supporting). **Siyuan Hong**: methodology (supporting). **Jie Wang**: data curation (supporting); methodology (supporting). **Chen Wang**: data curation (supporting). **Jingyi Shen**: data curation (supporting). **Wei Kong**: resources (supporting). **Guolin Zheng**: resources (supporting). **Jun Yan**: conceptualization (lead); data curation (lead); methodology (lead); writing—review and editing (supporting). **Jie Wu**: conceptualization (lead); methodology (lead); supervision (lead). **Changxi Zheng**: conceptualization (lead); formal analysis (lead); funding acquisition (lead); methodology (lead); resources (lead); writing—original draft (lead).

## Supporting information

Supplementary Material

## Data Availability

The data that support the findings of this study are available on request from the corresponding author. The data are not publicly available due to privacy or ethical restrictions.
