# Correction to “Air‐Stable Lithiation of MoS_2_ for Direct‐Bandgap Multilayers”

**DOI:** 10.1002/smsc.70310

**Published:** 2026-05-20

1

Qi Fu, Yichi Zhang, Jichuang Shen, Siyuan Hong, Jie Wang, Chen Wang, Jingyi Shen, Wei Kong, Guolin Zheng, Jun Yan, Jie Wu, Changxi Zheng. Air‐stable lithiation of MoS_2_ for direct‐bandgap multilayers. *Small Sci*. 2025: 5, 2500186. https://doi.org/10.1002/smsc.202500186

The Acknowledgments section of the original article has been revised to include an additional funding acknowledgement:

“This work was supported by Zhejiang Provincial Natural Science Foundation of China (grant no. XHD23A2003 to C.Z.), National Key R&D Program of China (grant nos. 2023YFA1406400 and 2024YFA1408102 to J.W.), National Natural Science Foundation of China (grant nos. 12174319 to C.Z. and 12174318 to J.W.), Research Center for Industries of the Future (RCIF project no. WU2023C001 to J.W. and C.Z.) at Westlake University, the China Postdoctoral Science Foundation (grant no. 2022M712844 to S.W.).”

We apologize for this error.

