# Supplementary material for: Air‐Stable Lithiation of MoS2 for Direct‐Bandgap Multilayers
Source: Small Sci. 2025 Jun 23;5(9):2500186. doi: 10.1002/smsc.202500186 (PMC12412521; doi:10.1002/smsc.202500186)
Supplement: Supplementary file 1 — Supplementary Material [file SMSC-5-2500186-s001.pdf]

Table of Content

Figure S1. Optical spectra measurements.

Figure S2. Calibration of AFM tip surface potential using an Au-Si-Al standard sample.

Figure S3. PFM lithography pattern and surface morphology.

Figure S4. Comparison of bare LICGC surface before and after PFM lithography.

Figure S5. Evolutions of the surface potentials of the two vicinal regions with and without PFM lithography, respectively.

Figure S6. Voltage maps of chess board and bars for PFM lithography.

Figure S7. Air stability of Li doped MoS_2 electronics.

Figure S8. Voltage maps of a logo for PFM lithography.

Figure S9. Height profile of the MoS_2 flake before and after Li intercalation.

Figure S10. Optical and surface topography image of MoS_2 flake before and after Li intercalation.

Figure S11. Low temperature (2K) PL.

Figure S12. Schematic of PFM lithography with negative voltage.

Figure S13. I-V curve measurements of an Al_2O_3/MoS_2/LICGC device.

Figure S14. Continuous current mapping during Li ions intercalation in multilayer MoS_2 ($V_{sample} = -10\ V$) for three times.

Figure S15. Schematic of the Li intercalation in multilayer MoS_2 with PFM lithography.

Figure S16. Raman spectrum of MoS_2 before and after modulation.

Figure S17. Transmission Electron Microscope (TEM) image of the MoS_2 flake before (a) and after (b) Li intercalation.

Section S1 Density functional theory (DFT)-calculated band structures of MoS_2.

Section S2 The estimation of charge doping level.

Section S3 The model of optical contrast before and after Li intercalation in MoS_2.

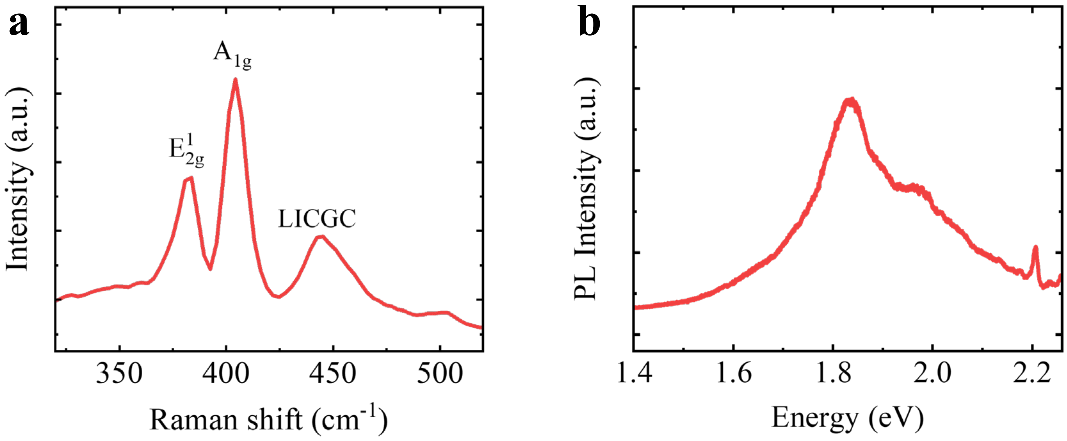

Figure S1. Optical spectra measurements. (a) Raman spectrum of monolayer MoS_2 channel of a device. (b) Photoluminescence spectrum of the monolayer MoS_2 channel.

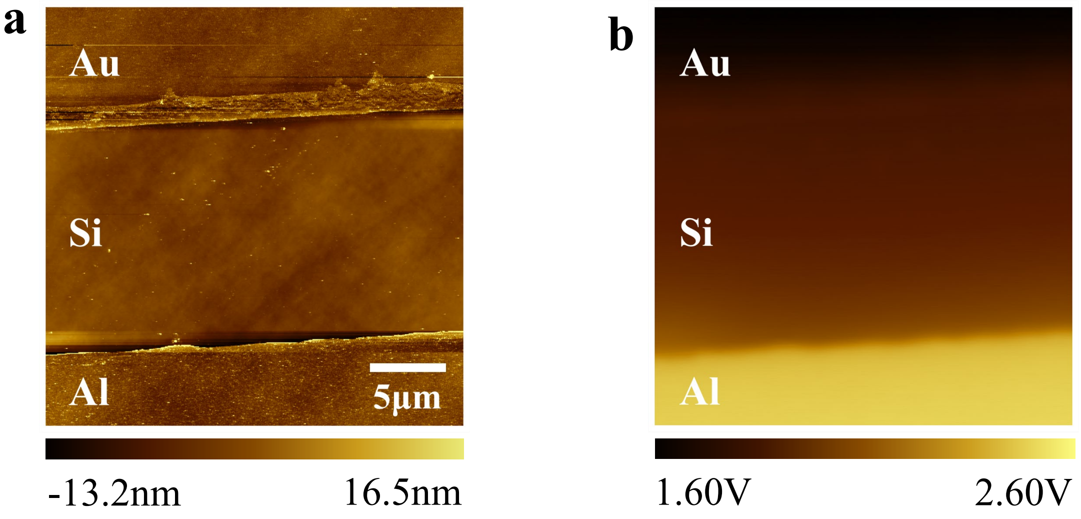

Figure S2. Calibration of AFM tip surface potential using an Au-Si-Al standard sample. (a) AFM topography of a Au-Si-Al calibration sample. (b) The corresponding KPFM image of the Au-Si-Al standard sample. The reference value of the fresh Au surface potential is 5.1 eV. According to the average surface potential of the Si and Al regions shown in (b), their calculated values are 4.815 eV and 4.295 eV, respectively.

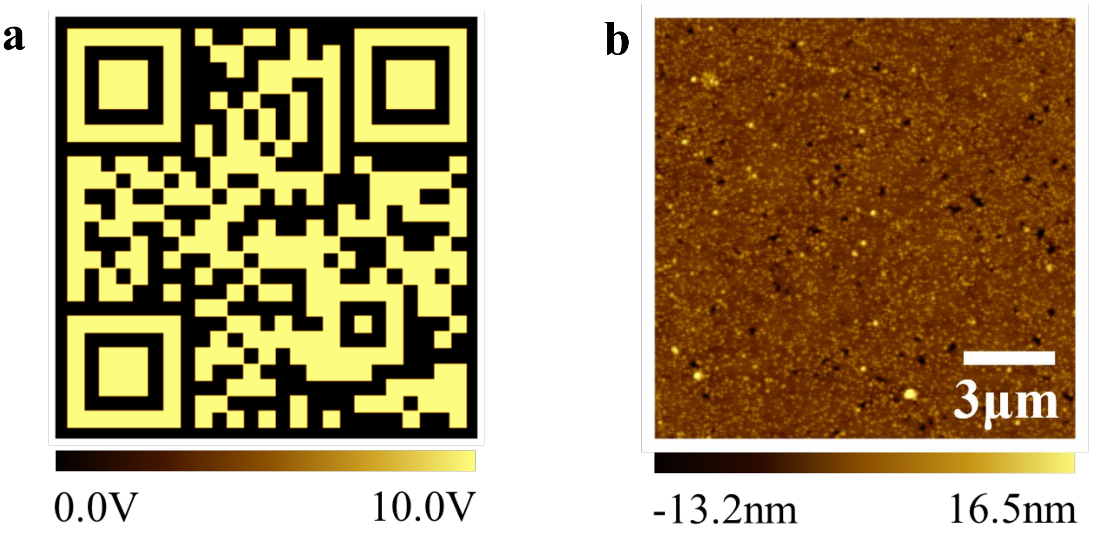

Figure S3. PFM lithography pattern and surface morphology. (a) The tip voltage map of a quick response (QR) code. (b) Corresponding AFM topography of the region on Al_2O_3/LICGC after PFM lithography. The change of surface morphology after PFM lithography does not happen.

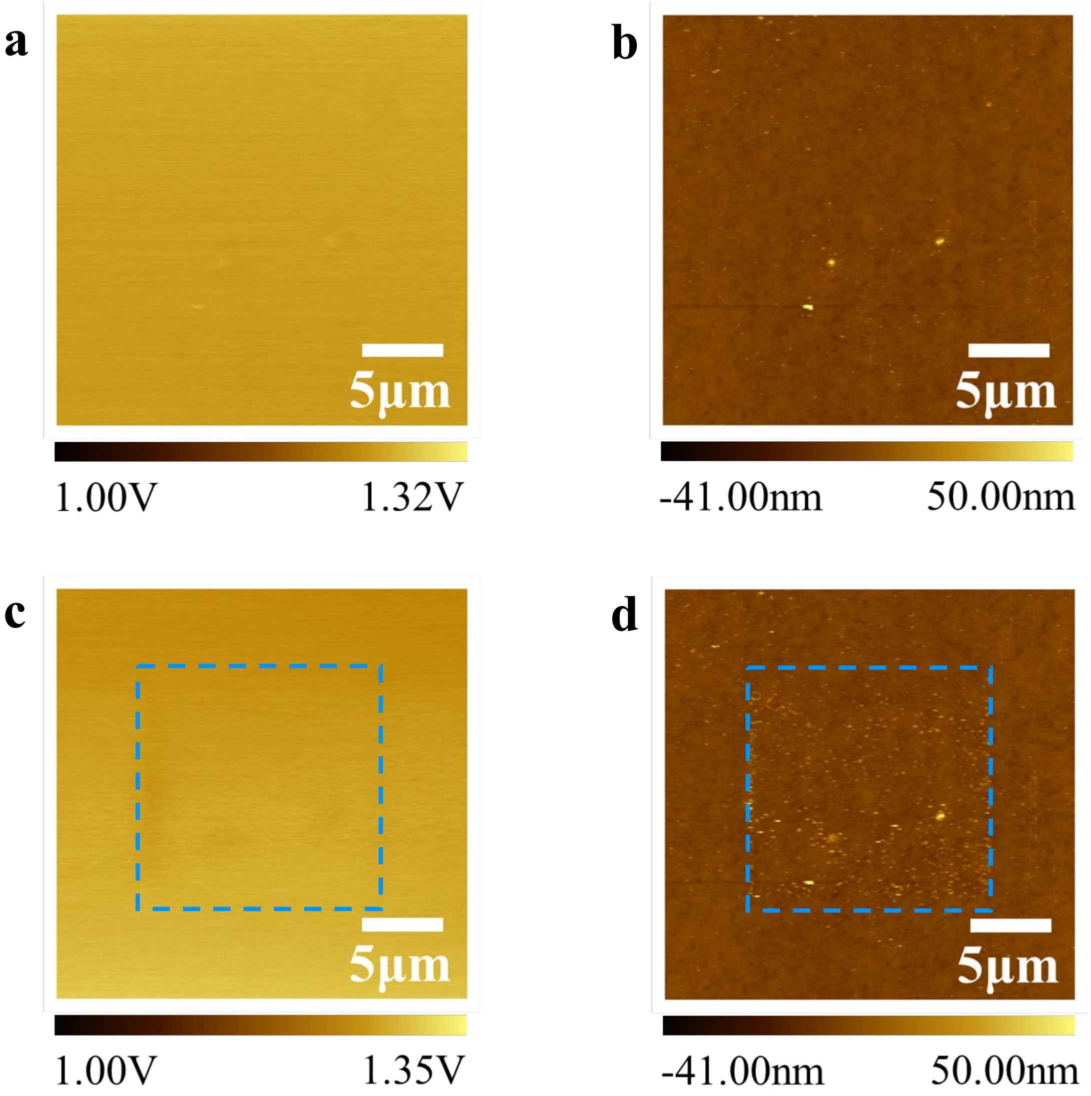

Figure S4. Comparison of bare LICGC surface before and after PFM lithography. (a) KPFM image of the bare LICGC surface before PFM lithography. (b) Corresponding AFM topography of the region. (c) KPFM image of the same region after PFM lithography. The dashed blue square indicates the region written by 5 V PFM lithography. No surface potential difference is observed. (d) Corresponding AFM topography of the same surface. The topography of the region after PFM lithography remains similar except some small dots emerge.

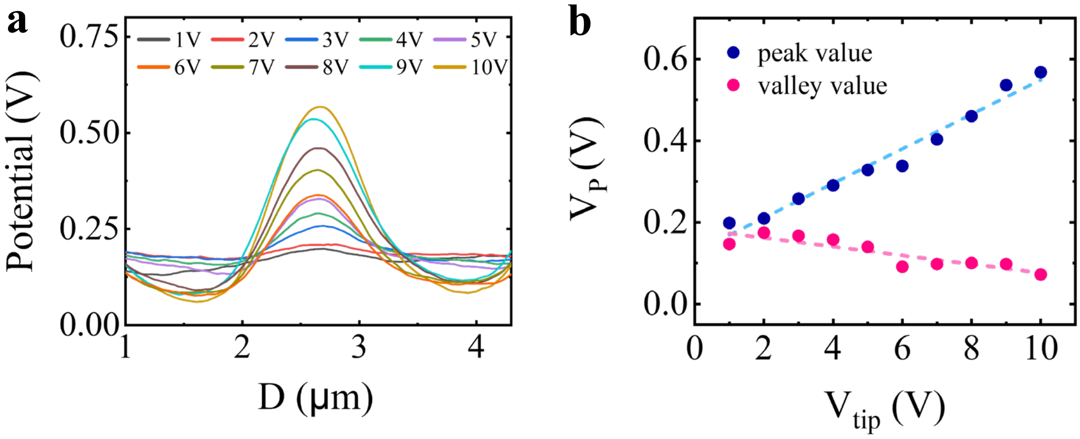

Figure S5. Evolutions of the surface potentials of the two vicinal regions with and without PFM lithography, respectively. (a) Surface potential curves of a single PFM lithography pattern and the regions nearby. The curves are extracted from Fig. 2c. (b) Evolution of peak value and valley value as a function of tip voltage. The peak values and valley values are taken from the peaks and the left valleys shown in (a).

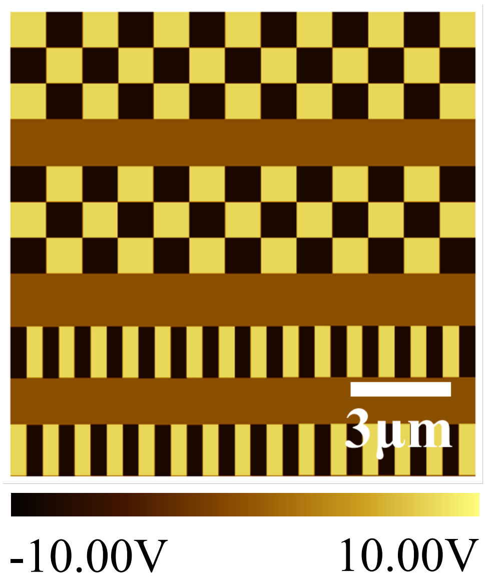

Figure S6. Voltage maps of chess board and bars for PFM lithography.

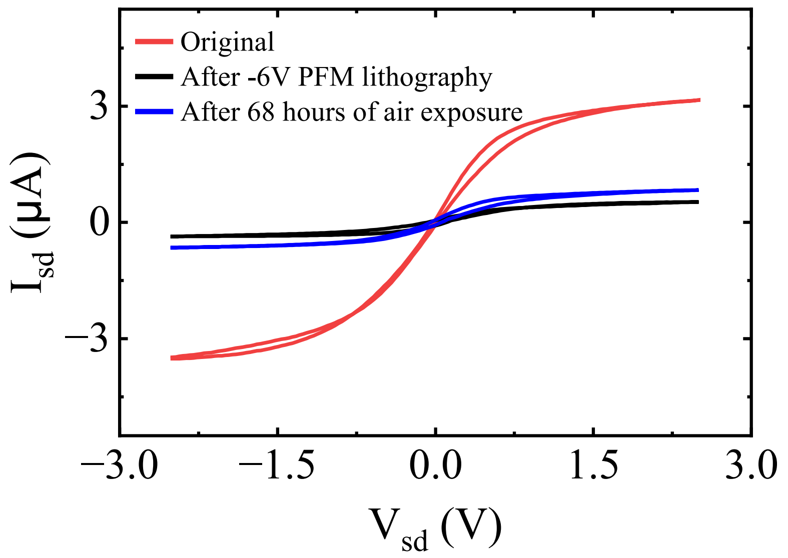

Figure S7. Air stability of Li doped MoS_2 electronics. I-V curves taken from an Al_2O_3/1L-MoS_2/LICGC device at three different stages in a series of original, after -6 V PFM lithography, and after 68 hours of air exposure.

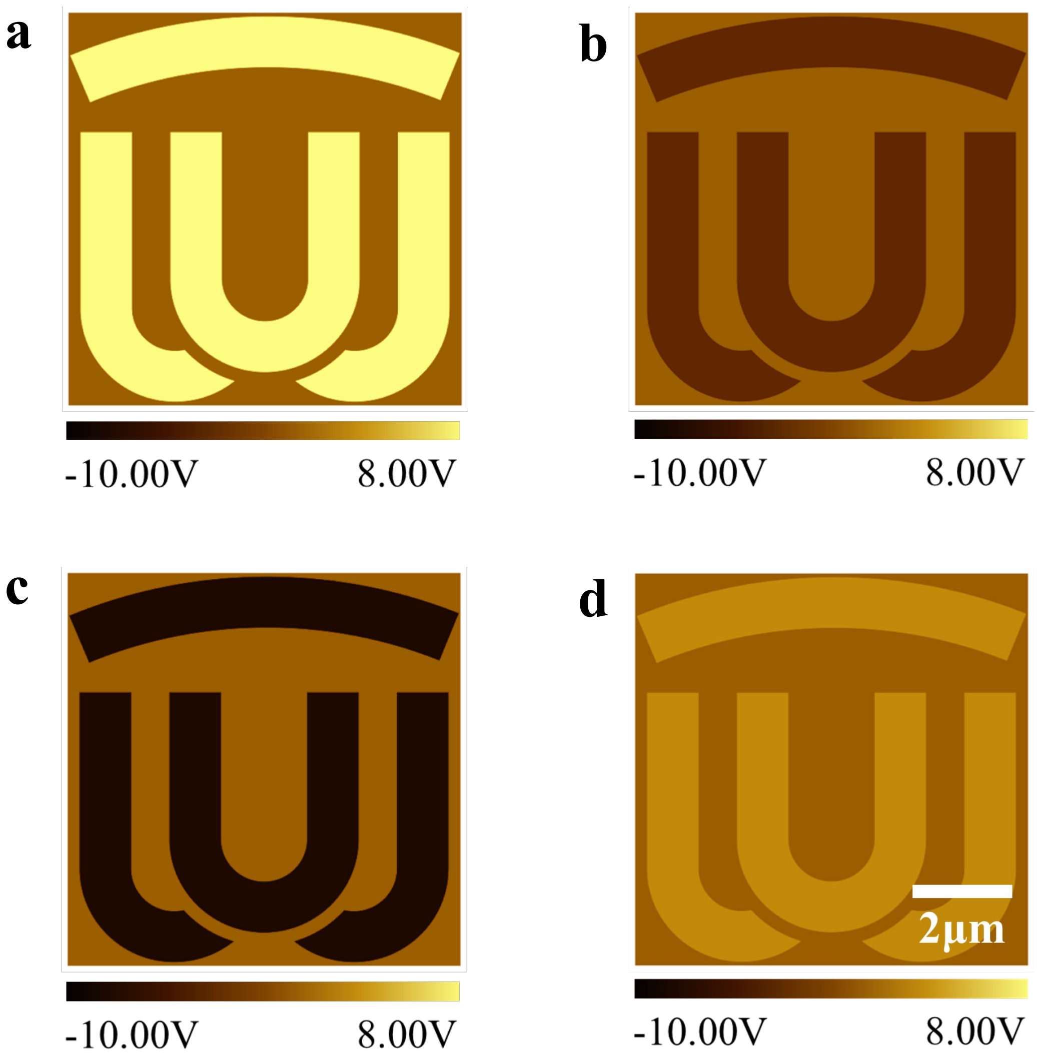

Figure S8. Voltage maps of a logo for PFM lithography. (a) Positive voltage map for writing dopant. (b) Negative voltage map for erasing dopant. (c) Negative voltage map for writing dopant. (d) Positive voltage map for erasing dopant.

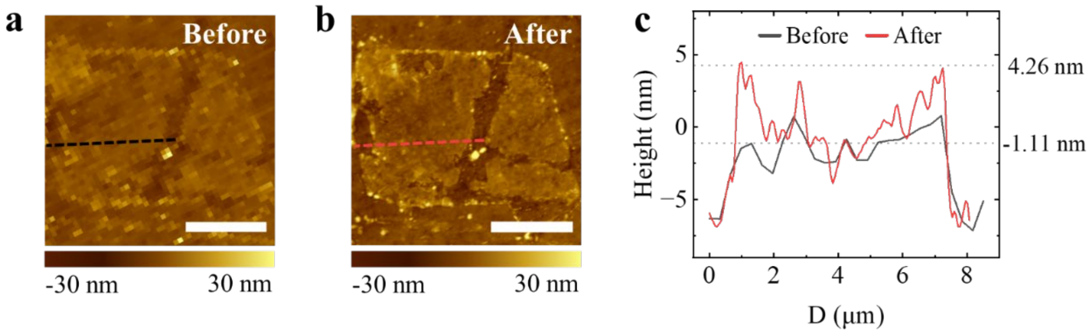

Figure S9. Height profile of the MoS_2 flake before and after Li intercalation. (a) AFM morphology image before Li intercalation (scale bar: 5 μm). (b) AFM morphology image after Li intercalation (scale bar: 5 μm). (c) Height line profile along the dash line in (a-b).

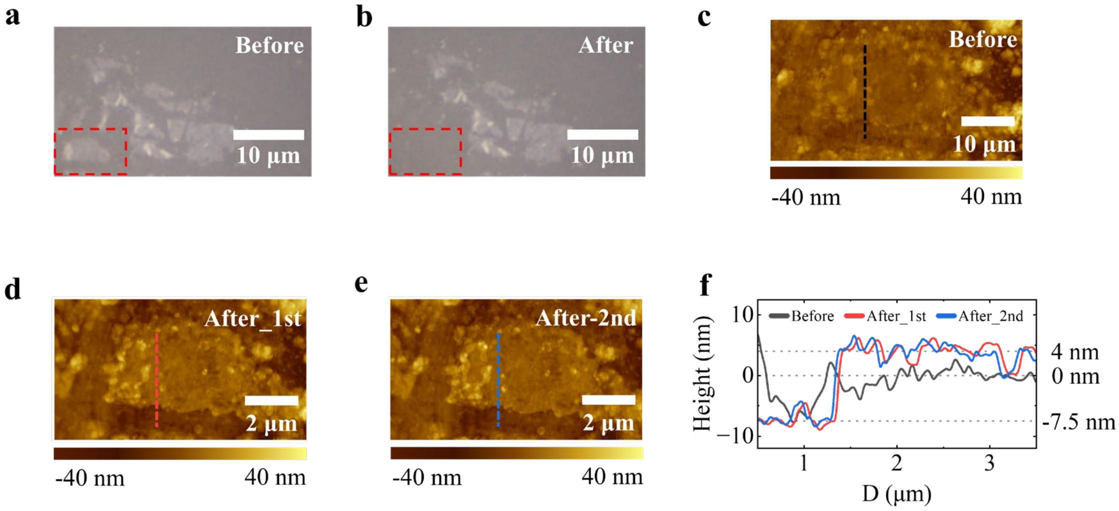

Figure S10. Optical and surface topography image of MoS_2 flake before and after Li intercalation. (a-b) Optical images of MoS_2 flakes before (a) and after (b) Li intercalation. (c-e) AFM surface morphology images before (c) and after (d-e) Li intercalation through PFM lithography. (f) Height line profile along the line in (c-e).

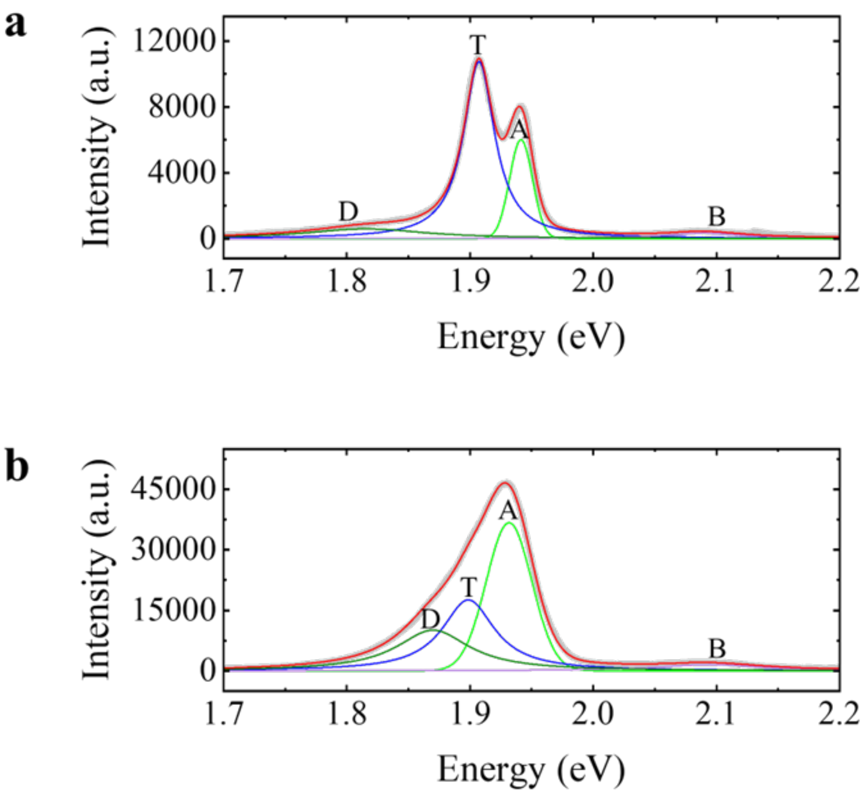

Figure S11. Low temperature (2K) PL. (a) From an exfoliated monolayer MoS_2 sample sandwiched between hexagonal boron nitride on Si/SiO_2. (b) From Li interacted Al_2O_3/MoS_2/LICGC device. Thick gray traces are experimental data, and smooth colored curves are multi-peak fittings: neutral (A) excitons (light green curve), trions (blue curve), B excitons (purple curve), disorder-trapped excitons (dark green curve), and the sum of those multi-peak fitting curves (red curve). For the as-made monolayer MoS_2, the spectra weight of trion is higher than that of exciton, while for the Li modulated multilayer MoS_2, the two spectral weights are comparable. The exciton-trion splitting is 34 meV for (**a**), and 33 meV for (b). Both observations indicate that the electron doping level of our Li intercalated device is not more than the as-made monolayer MoS_2. From the trion binding energy of 18 meV, we deduce that the Fermi energy in our direct gap device is about 15 meV.

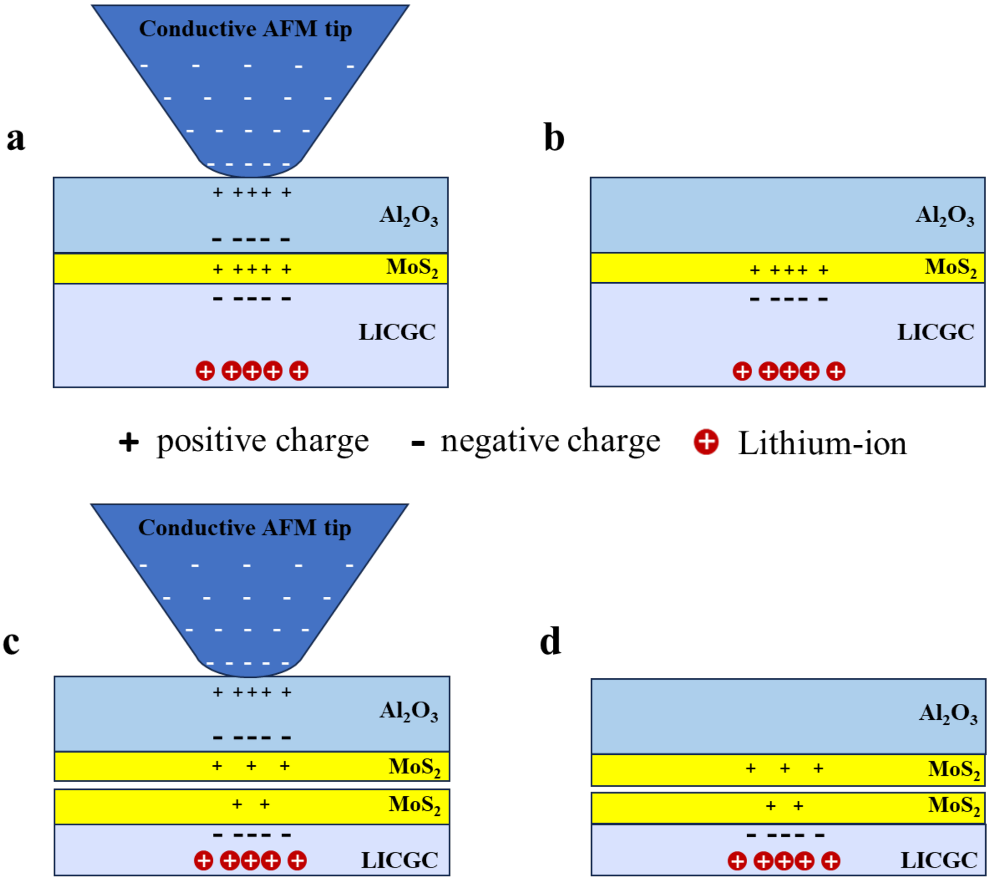

Figure S12. Schematic of PFM lithography with negative voltage. (a), (b), Models for Al_2O_3/MoS_2/LICGC device with monolayer MoS_2. (c), (d), Models for Al_2O_3/MoS_2/LICGC device with multilayer MoS_2.

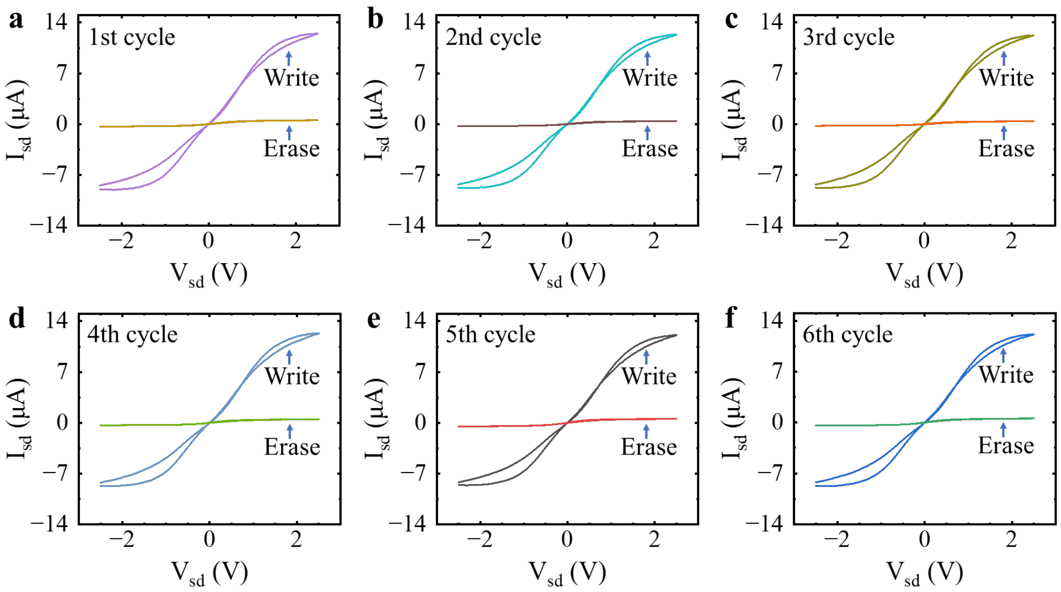

Figure S13. I-V curve measurements of an Al_2O_3/MoS_2/LICGC device, showing two resistance states switched by PFM lithography applied to the channel for 6 cycles.

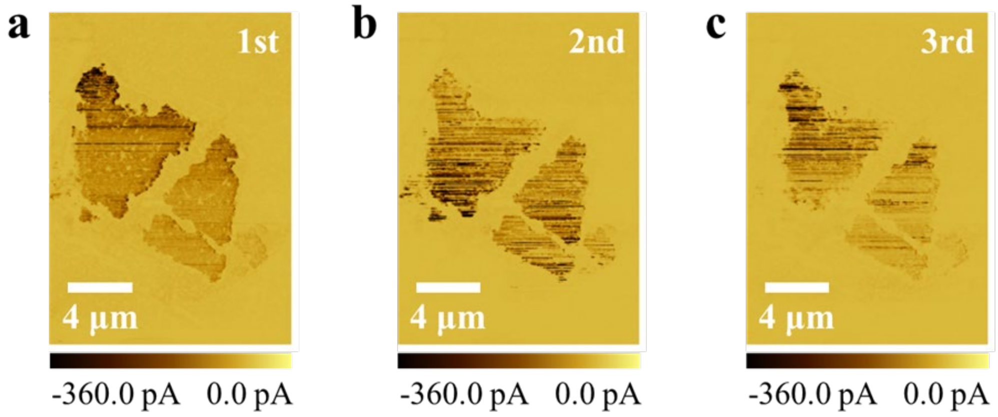

Figure S14. Current mapping during Li ions intercalation in multilayer MoS_2 ($V_{sample} = -10\ V$) over three trials. (a) First trial, (b) second trial, (c) third trial.

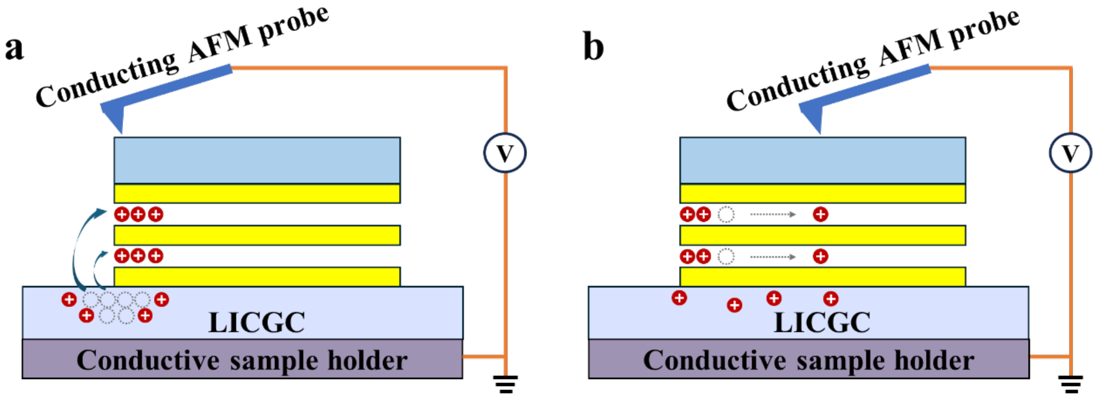

Figure S15. Schematic of Li intercalation in multilayer MoS_2 via PFM lithography. (a) Edge of the flake and (b) middle of the flake.

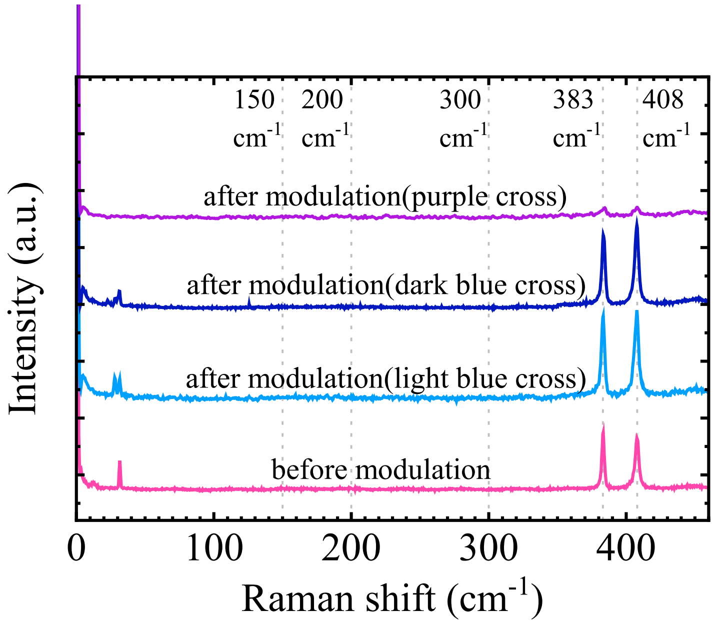

Figure S16. Raman spectrum of MoS_2 before and after modulation. (The correspondence between the spectrum color and position is completely consistent with that in Figure 4h of the manuscript.)

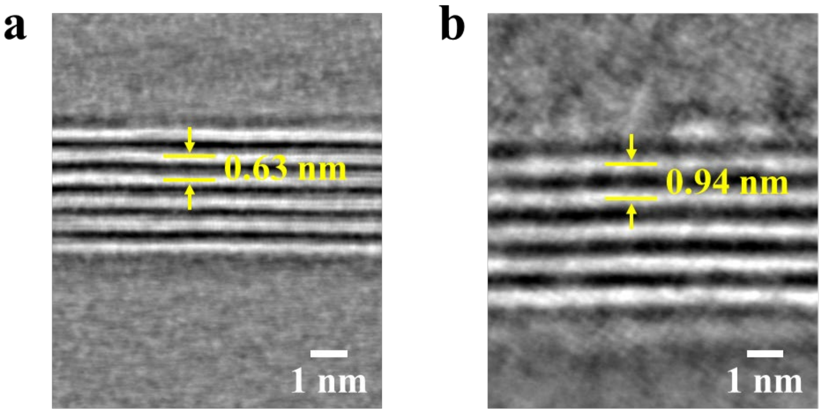

Figure S17. Transmission electron microscope (TEM) image of the MoS_2 flake before (a) and after (b) Li intercalation. The interlayer spacing of the MoS_2 flake is 0.63 nm and 0.94 nm before and after Li intercalation, respectively.

Section S1 Density functional theory (DFT)-calculated band structures of MoS_2.

The first-principle DFT calculations were performed using the Vienna Ab lnitio Simulation Package (VASP)[1]. The exchange and correlation functional was treated using Perdew-Burke-Ernzerhof (PBE) generalized gradient approximation (GGA)[2], and the plane-wave cut-off was set to 700 eV. The DFT-D3 method of Grimme with zero-damping function was employed to describe the vdW interaction correctly. A Γ-centered 15 × 15 × 1 k-points mesh was used for the self-consistent calculations of related structure. A series of different interlayer spacing parameters of 2H-MoS_2 with and without intercalation were manually set to observe their influence on the band structure after achieving fully self-consistent field (SCF) convergence.

It should be noted that our current modeling framework and parameters encounter difficulties in simulating a direct band gap using DFT calculations with intercalated Li atoms. We therefore use helium (He) atoms instead of Li atoms because He atoms have a size close to Li atoms. In addition, our Raman and PL results indicate that the doping level of Li-intercalated MoS_2 is low and that the structure remains in the 2H phase rather than the 1T phase. Thus, we believe that intercalated He atoms can serve as a good approximation.

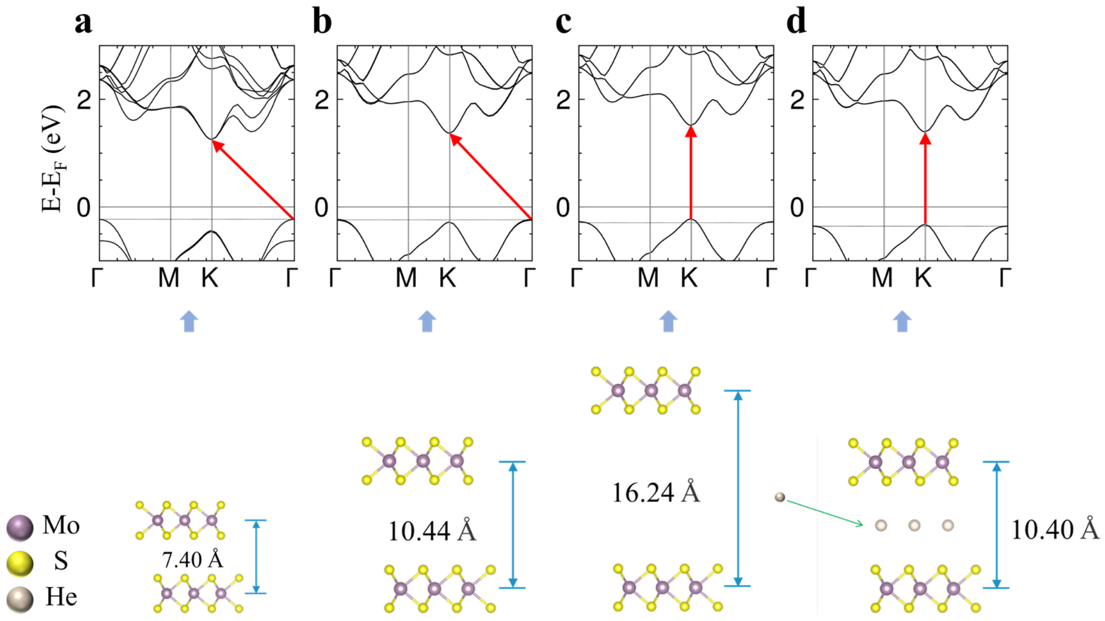

Figure S18. Density functional theory (DFT)-calculated band structures of MoS_2 under varying interlayer spacings with and without intercalation. Figure (a-c) illustrate the direct bandgap transition induced by interlayer spacing change without intercalation. Figure (d) shows that the interlayer spacing change required for the direct bandgap transition is significantly reduced with He intercalation. The numerical values of interlayer spacing for the MoS_2 structure in DFT calculations has been indicated in Figure (a-d).

Section S2 The estimation of charge doping level.

As indicated in Figure S11, the Fermi level under n-type doping was estimated to be approximately 15 meV based on low-temperature photoluminescence (PL) measurements. To estimate the system's carrier concentration, a simplified estimation can be derived from the relationship between the Fermi level and the two-dimensional electron gas (2DEG) carrier density.

For a two-dimensional parabolic band structure:

$$E(k_F) = \frac{\hbar^2 k_F^2}{2m^*}$$

In k-space, each k-point accommodates two spin states, yielding the density of states (DOS):

$$g(E) = \frac{m^*}{\pi\hbar^2}$$

The carrier concentration n_{2D} can be expressed as:

$$n_{2D} = \int_0^{E_F} g(E)\, dE = \int_0^{E_F} \frac{m^*}{\pi\hbar^2}\, dE = \frac{m^*}{\pi\hbar^2} \times E_F$$

Assuming an effective mass $m^* = 0.5\, m_e$ for the MoS_2 monolayer[3], and applying this value to direct-bandgap multilayer MoS_2, the calculated carrier concentration is: $n_{2D} \approx 3.13 \times 10^{12}\ cm^{-2}$.

This demonstrates that under appropriate doping levels, multilayer MoS_2 could transition to a direct-bandgap semiconductor without structural phase transition. Excessive doping concentrations could induce a phase transition from the 2H to the 1T phase[4-6].

Section S3 The model of optical contrast before and after Li intercalation in MoS_2.

Considering the trilayer system of Al_2O_3/multilayer MoS_2/LICGC, the reflected light intensity can be written as[7-9]:

$$I = \left| \frac{r_1 e^{i(\Phi_1+\Phi_2)} + r_2 e^{-i(\Phi_1-\Phi_2)} + r_3 e^{-i(\Phi_1+\Phi_2)} + r_1 r_2 r_3 e^{i(\Phi_1-\Phi_2)}}{e^{i(\Phi_1+\Phi_2)} + r_1 r_2 e^{-i(\Phi_1-\Phi_2)} + r_1 r_3 e^{-i(\Phi_1+\Phi_2)} + r_2 r_3 e^{i(\Phi_1-\Phi_2)}} \right|^2$$

The relative indices of refraction r_i, the phase shifts due to changes in the optical path Φ_i, and the contrast before Li intercalation C_{Before} are as follows:

$$r_1 = \frac{\tilde{n}_0 - \tilde{n}_1}{\tilde{n}_0 + \tilde{n}_1}$$

$$r_2 = \frac{\tilde{n}_1 - \tilde{n}_2}{\tilde{n}_1 + \tilde{n}_2}$$

$$r_3 = \frac{\tilde{n}_2 - \tilde{n}_3}{\tilde{n}_2 + \tilde{n}_3}$$

$$\Phi_i = \frac{2\pi \tilde{n}_i d_i}{\lambda}$$

$$C_{Before} = \frac{I_{MoS_2} - I_{substrate}}{I_{MoS_2} + I_{substrate}} = C(\tilde{n}_1, d_2) = C(n_1, k_1, d_2)$$

Taking the multilayer MoS_2 under 400 nm light irradiation as an example (as the visible light spectrum is located within a wavelength range of 380 to 740 nm): light is incident perpendicularly from air (refractive index $n_0 = 1$) onto the Al_2O_3/multilayer MoS_2/LICGC trilayer system. the Al_2O_3 layer is described by thickness d_1 = 27 nm and λ-dependent refractive index $n_1(\lambda = 400\text{ nm}) = 1.7019$ with a real part only[10], the multilayer MoS_2 is described by thickness d_2 = 5 nm and λ-dependent refractive index $n_2(\lambda = 400\text{ nm}) = 3.72-3.25i$[9], and the substrate LICGC is described by thickness $d_3 = 2.5\times10^5$ nm and λ-dependent refractive index $n_3(\lambda = 400\text{ nm}) = 1.4$, which is similar to the value of typical oxide glass and ceramics[11].

Substituting the above parameters for calculation: the contrast of the multilayer MoS_2 before Li intercalation is: $C_{Before} \approx 0.493$。

Next, we calculated the contrast of the multilayer direct-bandgap MoS_2 system

after the Li intercalation, considering vertical incident light as well. The air gap is introduced between the MoS_2 layers. Due to the complexity of the system, we calculated the contrast C_{After} based on the Transfer Matrix Method[12, 13].

For two adjacent medium interfaces (where the refractive index of the current layer is n_j and that of the next layer is n_{j+1})

The relative indices of refraction r_i can be written as:

$$r_j = \frac{n_j - n_{j+1}}{n_j + n_{j+1}}$$

The relative indices of transmission t_j can be written as:

$$t_j = \frac{2n_j}{n_j + n_{j+1}}$$

The phase shifts due to changes in the optical path β_j:

$$\beta_j = \frac{2\pi n_j d_j}{\lambda}$$

The propagation of light in a single layer (such as the j-th layer) is described by a 2×2 transfer matrix:

$$\mathrm{M}_j = \frac{1}{t_j}\begin{bmatrix} e^{i\beta_j} & r_j e^{i\beta_j} \\ r_j e^{-i\beta_j} & e^{-i\beta_j} \end{bmatrix}$$

The Cascaded Transfer Matrix is obtained by the matrix multiplication of the individual matrices:

$$\mathrm{M}_{\mathrm{total}} = \prod_{j=1}^{N} \mathrm{M}_j$$

The reflected light intensity can be written as:

$$I = |r_{\mathrm{total}}|^2 = \left|\frac{M_{21}}{M_{11}}\right|^2$$

The contrast of the multilayer MoS_2 after Li intercalation is:

$$C_{After} = \frac{I_{MoS_2} - I_{substrate}}{I_{MoS_2} + I_{substrate}} = C(\tilde{n}_1, d_2) = C(n_1, k_1, d_2)$$

According to the supplementary data shown in Figure S9, the Li intercalation into the multilayer MoS_2 with a thickness of 5 nm (~8 layers) results in an approximate increase of 5 nm in thickness at the edges, thus introducing an air layer with a thickness

of 0.71 nm, the monolayer MoS_2 is described by thickness $d_{MoS_2} = 0.63\ nm$ and λ-dependent refractive index n_2(λ=400 nm) = 2.39-2.23i[9], the remaining material parameters are consistent with those of the previous trilayer system. By calculating we can get the contrast of the multilayer MoS_2 after Li intercalation is: $C_{After} \approx -0.020$.

In summary, based on the current model, we conducted a preliminary analysis of the change in optical contrast trends before and after the Li intercalation of multilayer MoS_2 system. The calculation results indicate that the MoS_2 optical contrast after Li intercalation exhibits a significant decreasing trend from 0.493 to -0.020 under the illumination of incident light at 400 nm, which is consistent with the phenomena we observed in experiments (Figure S18).

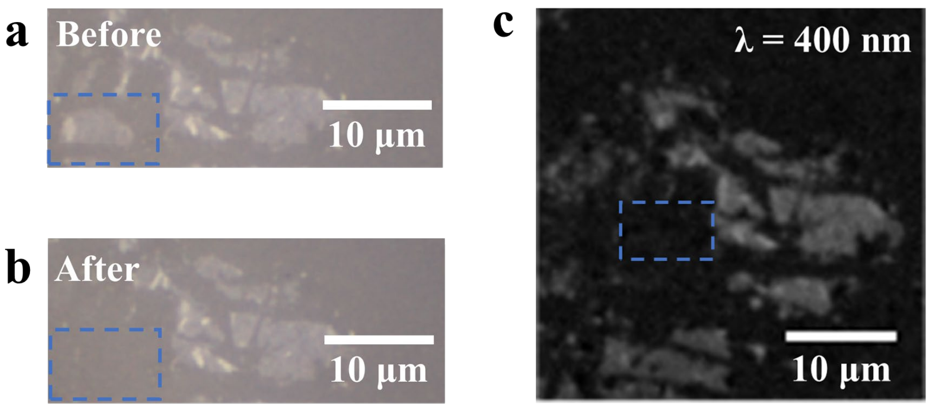

Figure S19. Optical images. The MoS_2 sample before and after Li intercalation imaged with white light (a-b), and the same area imaged with purple light ($\lambda = 400\ nm$) (c). The MoS_2 flakes without Li intercalation are clearly visible in (a-c).

References

(1) Kresse, G.; Furthmuller, J., Efficient iterative schemes for ab initio total-energy calculations using a plane-wave basis set, 1996, Physical Review B 54 (16), 11169-11186, https://doi.org/10.1103/PhysRevB.54.11169

(2) Perdew, J. P.; Burke, K.; Ernzerhof, M., Generalized gradient approximation made simple, 1996, Phys Rev Lett 77 (18), 3865-3868, https://doi.org/10.1103/PhysRevLett.78.1396

(3) Yu, S.; Xiong, H. D.; Eshun, K.; Yuan, H.; Li, Q. L., Phase transition, effective mass and carrier mobility of MoS_2 monolayer under tensile strain, 2015, Appl Surf Sci 325, 27-32, https://doi.org/10.1016/j.apsusc.2014.11.079

(4) Xiong, F.; Wang, H. T.; Liu, X. G.; Sun, J.; Brongersma, M.; Pop, E.; Cui, Y., Li Intercalation in MoS_2: In Situ Observation of Its Dynamics and Tuning Optical and Electrical Properties, 2015, Nano Lett 15 (10), 6777-6784, https://doi.org/10.1021/acs.nanolett.5b02619

(5) Xia, J.; Wang, J.; Chao, D. L.; Chen, Z.; Liu, Z.; Kuo, J. L.; Yan, J. X.; Shen, Z. X., Phase evolution of lithium intercalation dynamics in 2H-MoS_2, 2017, Nanoscale 9 (22), 7533-7540, https://doi.org/10.1039/c7nr02028g

(6) Costanzo, D.; Jo, S.; Berger, H.; Morpurgo, A. F., Gate-induced superconductivity in atomically thin MoS_2 crystals, 2016, Nat Nanotechnol 11 (4), 339-+, https://doi.org/10.1038/Nnano.2015.314

(7) Blake, P.; Hill, E. W.; Castro Neto, A. H.; Novoselov, K. S.; Jiang, D.; Yang, R.; Booth, T. J.; Geim, A. K., Making graphene visible, 2007, Appl Phys Lett 91 (6), https://doi.org/10.1063/1.2768624

(8) Hsu, C. W.; Frisenda, R.; Schmidt, R.; Arora, A.; de Vasconcellos, S. M.; Bratschitsch, R.; van der Zant, H. S. J.; Castellanos-Gomez, A., Thickness-Dependent Refractive Index of 1L, 2L, and 3L MoS_2, $MoSe_2$, WS_2, and WSe_2, 2019, Adv Opt Mater 7 (13), https://doi.org/10.1002/adom.201900239

(9) Zhang, H.; Ma, Y. G.; Wan, Y.; Rong, X.; Xie, Z.; Wang, W.; Dai, L., Measuring the Refractive Index of Highly Crystalline Monolayer MoS_2 with High Confidence, 2015,

Sci Rep-Uk 5, https://doi.org/10.1038/srep08440
(10) Boidin, R.; Halenkovic, T.; Nazabal, V.; Benes, L.; Nemec, P., Pulsed laser deposited alumina thin films, 2016, Ceram Int 42 (1), 1177-1182, https://doi.org/10.1016/j.ceramint.2015.09.048
(11) Yamada, H.; Takemoto, K., Local structure and composition change at surface of lithium-ion conducting solid electrolyte, 2016, Solid State Ionics 285, 41-46, https://doi.org/10.1016/j.ssi.2015.08.019
(12) Katsidis, C. C.; Siapkas, D. I., General transfer-matrix method for optical multilayer systems with coherent, partially coherent, and incoherent interference, 2002, Appl Optics 41 (19), 3978-3987, https://doi.org/10.1364/Ao.41.003978
(13) Benamira, A.; Pattanaik, S., Application of the Transfer Matrix Method to Anti-reflective Coating Rendering, 2020, Lect Notes Comput Sc 12221, 83-95, https://doi.org/10.1007/978-3-030-61864-3_8
